# Identifying experts in the field of visual arts using oculomotor signals

**DOI:** 10.16910/jemr.11.3.3

**Published:** 2018-05-24

**Authors:** Marcin Kołodziej, Andrzej Majkowski, Remigiusz J. Rak, Piotr Francuz, Paweł Augustynowicz

**Affiliations:** Warsaw University of Technology, Poland; John Paul II Catholic University of Lublin, Poland

**Keywords:** Expert system, eye-tracking, fixation, clusters, neural network, support vector machine

## Abstract

In this article, we aimed to present a system that enables identifying experts in the field of visual art based on oculographic data. The difference between the two classified groups of tested people concerns formal education. At first, regions of interest (ROI) were determined based on position of fixations on the viewed picture. For each ROI, a set of features (the number of fixations and their durations) was calculated that enabled distinguishing professionals from laymen. The developed system was tested for several dozen of users. We used k-nearest neighbors (k-NN) and support vector machine (SVM) classifiers for classification process. Classification results proved that it is possible to distinguish experts from non-experts.

## Introduction

In the field of empirical esthetics, we pose questions about the differences between experts and non-experts in terms of their esthetic preferences and emotional, behavioral, or neurophysiological reactions. In the vast majority of them, we assume that the experts in the field of art (as opposed to the laymen) are continuing or completed their studies in art history, an academy of fine arts, or a conservatory. We assume that they are involved in some kind of art or actively participate in cultural life (e.g., they visit museums and exhibitions, paint, take photographs, sculpture, or read about art either professionally or as a hobbyist. Furthermore, studies have shown inhomogeneity of groups of experts and non-experts confronted with evaluation of works of art. Therefore, there is a need to look for an objective method of measuring expert level in the field of art.

Oculography, as a method of measuring of human visual activity, gives some possibilities in this field. One of the reliable indicators of an interest in a specific fragment of an image is the density of fixations, registered by eye-tracker [ 1 ]. Regions of interest (ROI) are interpreted as places of especially high information values [ 2-5 ]. Generally, higher values of many oculomotor indicators (e.g., average fixation time, duration of fixations or length of saccades preceding fixations) are recorded in areas of high information values [ 6, 7, 8, 9, 10, 11 ]. The results of eye-tracking research to find experts and non-experts in the field of visual arts show some differences in the distribution of fixations on the known and unknown pictures [ 1 ]. Practicing artists often pay attention to these fragments of images that lie beyond the obvious centers of interest (e.g., the faces of people) unlike non-experts. It was also found that the experts have a more global strategy to search image area than non-experts [ 12 ]. However, non-experts pay more attention to objects and people shown in pictures, whereas experts are more interested in structural features of these images. Vogt and Magnussen [ 13 ] found that the non-experts fix their sight longer on earlier watched parts of images than the experts. It was also found that non-experts, regardless of the type of task being performed (free viewing of photos or scanning them to find the specified object) fix their sight according to the salience-driven effect, which is in line with the bottom up strategy of information processing [ 14 ]. Hermens [ 15 ] presented an extensive review of the literature concerning eye movements in surgery. On the basis of eye movements some techniques to assess surgical skill were developed, and the role of eye movements in surgical training was examined. Sugano [ 16 ] investigated the possibility of image preference estimation based on a person’s eye movements. Stofer and Che [ 17 ] investigated of expert and novice meaning-making from scaffolded data visualizations using clinical inter-views. Boccignone [ 18 ] applied machine learning to detect expertise from the oculomotor behavior of novice and expert billiard players.

Viewing a picture runs fragmentarily. While viewing a picture, people focus their eyes on different parts of it with different frequencies [ 2 ]. If an image is watched by a dozen or so people, it is likely that they will pay attention to its similar fragments. This tendency has been previously confirmed by numerous studies, started from experiments conducted by the pioneers of oculography such as [ 6,19, 20, 21 ]. Can we, based on the coordinates and durations of fixations, predict who is watching the image: an expert or a layman? In this article, we present a system that enables identifying experts in the field of art based on eye movements while watching assessed paintigs. The difference between the classified groups of people concerns formal education and related to it greater or lesser experience in dealing with works of art.

## Participants and setup

In this study, we collected data from 44 people: 23 experts (including 11 women) and 21 non-experts (including 11 women), who were in the age group of 20–27 years (mean value = 23.4; standard deviation = 1.6). Eighty-five percent of the people in the group of experts were students of the fourth and fifth years of studies, and fifteen percent were students of the second and third year, mainly art history (90%) and painting and graphics (10%). In addition to formal education, all of them declared interest in visual arts and about half of them have been actively involved in some form of art (painting, graphics, sculpture, photography, design, etc.) for several years. Non-experts did not meet any of the above criteria. All persons had normal or corrected to normal vision and did not report any symptoms of neurological disorder. All people participating in the experiments received financial compensation.

We used digitized reproductions of five known paintings. The list of the images is presented below:

P1.  James J. Tissot—The Traveller [1883–1885]),

P2.  Caravaggio—Crucifixion of St. Peter [1600],

P3.  Gustave Courbet—Malle Babbe [1628–1640],

P4.  Carl Holsøe—Reflections [year unknown],

P5.  Ilja Repin—Unexpected Visitor [1884–1888]).

One image was used in the instructions for users:

P0.  Alexandre Cabanel—Cleopatra Testing Poisons on Condemned Prisoners [1887].

In this study, we used SMI RED 500 eye-tracker. The images were displayed on a color monitor with 1920x1200 pixel resolution. The person being examined was sitting in front of a monitor at a distance of approximately 65 cm. The program for stimuli presentation and recording the reactions of the respondents was written in E-Prime v.2.0. The subjects answered the question of esthetic evaluation using a keyboard with a variable arrangement of keys.

The task of the users was to watch random sequence of five test pictures. Their eye movements were recorded while they were viewing the images, in the form of fixations and fixation durations. The recordings lasted for approximately 20 min, including the time required for calibration of the eye-tracker and passing instructions to the user. The experiment consisted of the following phases:

1. Instruction on how to perform tasks in test phase,

2. Eye-tracker calibration,

3. The draw of the image,

4. Watching image for 15 s,

5. Esthetic image evaluation.

It needs to be highlighted that our aim was not classify experts and non-experts based on their aesthetic preferences. The idea was to check whether we can distinguish experts and non-experts from the way they view the images.

## Methods

**Figure 1:**
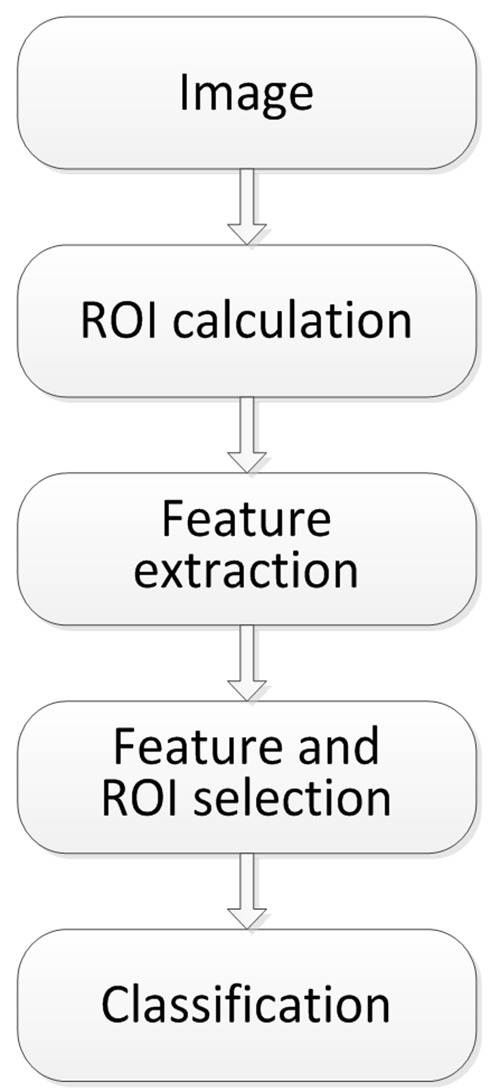
The steps of operation of the system for automatic recognition of experts

We assumed that for images, there are individual ROIs, which in a different way attract the attention of experts and non-experts. Therefore, we specified sets of ROIs for each image separately. For each ROI, we calculated the following features: the number of fixations and the average duration of fixation that could enable distinguishing an expert from non-expert. We did not use diameter of eye pupil as a feature, because it is linked significantly with the brightness of the observed portion of the image [ 22,23 ]. We deliberately limited ourselves to static features related to specified clusters. We did not consider transition between clusters which might be useful [ 24 ]. We are aware that in this way we could limit the classification accuracy, but the purpose of the article was to check only static features. In the first step, the calculated features were used to learn the classifier. Then, the system was tested using cross-validation test (CV). Block diagram of the proposed system is given in Fig. 1.

### Specification of ROI

We considered several methods to specify ROI. The simplest of them included an arbitrary division of an image on separate areas (e.g., rectangles). However, in this case, both the selection of size and number of ROIs was a big problem. Consequently, we decided that such a simple division is unnatural and ineffective. Therefore, we used number of fixations to identify ROIs.

To specify ROIs, based on registered fixations, many clustering methods could be applied. The basis of most of them is the similarity between elements, expressed by that the observation x belongs to k-th cluster can be exsome metrics. Hierarchical methods, K-means, and fuzzy cluster analysis are frequently used for that purpose [ 8 ]. It turns out that, depending on the nature of observations, the type of method used plays an important role. Not without significance is the number of clusters, on which we want to divide the observations. In a number of known methods, the researcher must decide on the number of clusters. This makes analysis more difficult and requires a researcher participation in working out results.

We decided to use expectation-maximization (EM) clustering algorithm [ 4 ]. Bayesian information criterion (BIC) [ 9 ] was implemented to automatically determine the number of meaningful clusters. In EM algorithm, we used approximation of distribution of observations (x,y) with the use of mixtures probability density functions of normal distributions [ 10 ]. Suppose that the probability density function of observations x for K clusters is defined as [ 11 ]:

**(1):**
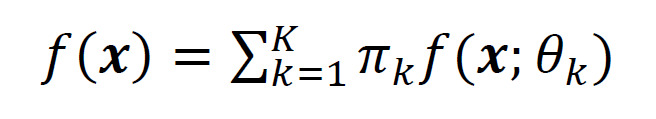

where f(x; Θ_k_) is a probability density function of the k-th cluster with Θ_k_ parameter and π_k_ depicts a mixture parameter. In case f(x; Θ_k_ ) is a normal distribution function, there exists Θ_k_ = (μ_k_ , 𝕽_k_ ), where μ_k_ is the vector of expected values for observations and 𝕽_k_ is the covariance matrix. We can use the EM algorithm to determine the expected values’ vectors and the covariance matrix of the probability density function of the k-th cluster. Let us define Ψ = {π_k_, Θ_k_: k = 1, … , K} as a set of parameters of normal distributions’ mixture. Then, the probability p_ik_ that the observation x belongs to k-th cluster can be expressed as [ 9 ]:

**(2):**
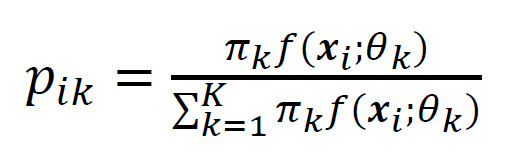

This is a basic step of EM method, denoted as E. In the following steps (called M), we can estimate the parameters of f(x) [ 22 ]:

**(3):**
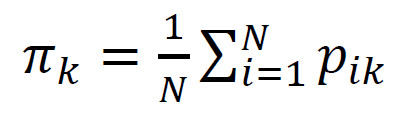

**(4):**
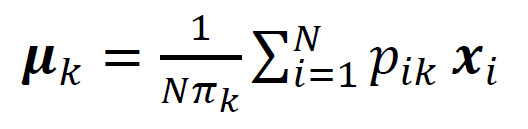

**(5):**
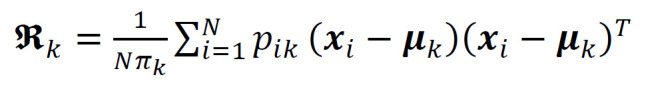

where N is the number of fixations. Using this procedure in an iterative mode, starting from an initial value of normal distribution and repeating steps E and M, we can guarantee that the logarithmic reliability of the observed data did not decrease [ 22 ]. This means that the parameters Θ_k_ converge to at least one of a local maximum of logarithmic likelihood function. It should be noted that an observation belongs to the k-th cluster, when the value p_ik_/p_k_ is the maximum, where p_k_ = ∑^N^_i=1_=p_ik_.

Clusters were determined for all registered fixations (for experts and non-experts), as large number of fixations ensured cluster calculation that can be interpreted as representative ROI.

### Feature extraction and selection

A fixation is described by its location on the screen (x,y) and/or its duration. Therefore, for each person and each cluster k (ROI), we determined features associated with fixations:

l_k_• – the number of fixations in k cluster,

t_k_• – the average fixation time in k cluster.

Consequently, we calculated 2K features (two features for each of clusters). Features were determined without data normalization (method labeled Z⁰) and for four different normalization methods (labeled Z¹, . . . , Z³), for which standardized z^j^_i_ values were calculated according to the general rule:

**(6):**
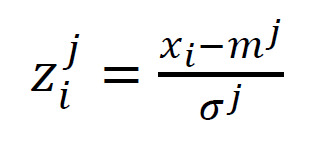

where x_i_-number of fixations or fixation duration, m-mean value and σ-standard deviation, j=1, 2 or 3 denote Z¹, Z² or Z³ normalization method. In the case of Z¹ − m¹ and σ¹ refer to all data together. In the case of Z² − m² and σ² refer to individual users. In the case of Z³ − m³ and σ³ refer to the individual users and viewed images. Thus, the z_i_³ takes into account individual differences between people examined separately for each image.

After feature extraction, the resulting features were assigned to specific ROIs. Not all features were equally useful for classification. Therefore, it seems sensible to make their selection. We decided to use two known feature selection methods: t-statistic [ 23 ] and sequential forward selection (SFS) [ 25 ]. The first ranking method allows to determine the best features for the purpose of distinguishing two classes. Having knowledge about the observations for experts and non-experts, we were able to compare feature distribution for each ROI. In this method, only statistical distribution of the features was used; the results of classification are not taken into consideration. Unfortunately, as a result of this method, we often obtained correlated features. In the second feature selection method—SFS, as a criterion, classification accuracy calculated for the tested features is used. Consequently, such selection generates features that are more independent.

### Classification

We used k-nearest neighbors classifier (k-NN) and support vector machine (SVM) with different types of kernel functions. K-NN classifier compares the values of the explanatory variables from the test set with the variables from the training set. K nearest observations from the training set were chosen. On the basis of this choice, classification is made. The definition of “nearest observation” boils down to minimizing a metric measuring the distance between two observation vectors. We applied the Euclidean metric. K-NN classifier is useful especially when the relationship between the explanatory and training variables is complex or unusual.

The essence of SVM method is separation of a set of samples from different classes by a hyperplane. SVM enables classification of data of any structure, not just linearly separable. There are many possibilities of determining the hyperplane by using different kernel functions, but the quality of the divisions is not always the same. Application of proper kernel function increases the chances of improving the separability of data and the efficiency of classification. In our experiments, we used linear kernel, sigmoid (MLP) kernel, and RBF kernel [ 11 ].

### Training and Testing

We decided not to use the same data at the training and testing stage. So, we implemented a leave-one-out test [ 11 ]. It is a modified cross-validation test (CV), when all N examples are divided into N subsets, containing one element. In our case, for testing, data from only one user was taken, whereas for training the classifier the data registered for all the other users was used. This procedure was repeated consecutively for all users, and then the classification accuracies were averaged. This approach ensures that classifier was tested and learned on separate data sets and subsequent averaging provided correct result.

## Results

The results comprise the classification accuracies for two classes: experts and non-experts. Classification accuracy was defined as the sum of true positives and true negatives divided by the number of all examples. Tables 1–7 include the classification accuracies for respective images (P1–P5) for the various methods of data standardization (Z⁰, Z¹–Z³). All details such as type of classifier, number of features, and feature selection method are given in the headers of tables. We used variable number of features (10, 5, and 3) selected using t-statistic or SFS for classification. The classification results presented in this study show that it is possible to distinguish an expert from non-expert using oculographic signals. We obtained the highest average classification accuracy for the SVM– MLP method for five best features and SFS selection method (Table 7).

**Table 1.** Classification accuracies for SVM-MLP method, 10-best features selected using t-statistic.

Picture	Z⁰	Z¹	Z²	Z³	mean
P1	0.75	0.89	0.64	0.67	0.74
P2	0.51	0.77	0.67	0.62	0.64
P3	0.57	0.54	0.74	0.71	0.64
P4	0.65	0.54	0.84	0.62	0.66
P5	0.73	0.73	0.78	0.63	0.72
mean	0.64	0.7	0.73	0.65	

**Table 2.** Classification accuracies for 3-NN method, 10-best features selected using t-statistic.

Picture	Z⁰	Z¹	Z²	Z³	mean
P1	0.75	0.75	0.78	0.81	0.77
P2	0.54	0.56	0.59	0.59	0.57
P3	0.34	0.6	0.6	0.6	0.54
P4	0.68	0.65	0.62	0.65	0.65
P5	0.65	0.55	0.58	0.55	0.58
mean	0.59	0.62	0.63	0.64	

**Table 3.** Classification accuracies for SVM-linear method, 10-best features selected using t-statistic.

Picture	Z⁰	Z¹	Z²	Z³	mean
P1	0.72	0.75	0.75	0.81	0.76
P2	0.69	0.77	0.64	0.56	0.67
P3	0.69	0.69	0.57	0.6	0.64
P4	0.78	0.65	0.68	0.7	0.70
P5	0.7	0.7	0.73	0.68	0.70
mean	0.72	0.71	0.67	0.67	

**Table 4.** Classification accuracies for SVM-RBF method, 10-best features selected using t-statistic.

Picture	Z⁰	Z¹	Z²	Z³	mean
P1	0.58	0.53	0.64	0.67	0.61
P2	0.62	0.64	0.44	0.49	0.55
P3	0.57	0.66	0.54	0.51	0.57
P4	0.62	0.65	0.59	0.62	0.62
P5	0.6	0.5	0.55	0.6	0.56
mean	0.59	0.59	0.55	0.57	

**Table 5.** Classification accuracies for SVM-MLP method, 5-best features selected using t-statistic.

Picture	Z⁰	Z¹	Z²	Z³	mean
P1	0.67	0.86	0.64	0.89	0.77
P2	0.51	0.46	0.51	0.77	0.56
P3	0.63	0.63	0.69	0.51	0.62
P4	0.57	0.57	0.7	0.81	0.66
P5	0.75	0.55	0.6	0.65	0.64
mean	0.62	0.61	0.62	0.72	

**Table 6.** Classification accuracies for SVM-MLP method, 3-best features selected using SFS.

Picture	Z⁰	Z¹	Z²	Z³	mean
P1	0.72	0.78	0.83	0.89	0.81
P2	0.72	0.74	0.59	0.69	0.69
P3	0.83	0.77	0.71	0.57	0.72
P4	0.7	0.76	0.76	0.62	0.71
P5	0.83	0.65	0.7	0.8	0.75
mean	0.76	0.74	0.72	0.71	

**Table 7.** Classification accuracies for SVM-MLP method, 5-best features selected using SFS.

Picture	Z⁰	Z¹	Z²	Z³	mean
P1	0.75	0.89	0.92	0.81	0.84
P2	0.72	0.51	0.79	0.72	0.69
P3	0.69	0.8	0.6	0.69	0.70
P4	0.62	0.65	0.76	0.81	0.71
P5	0.73	0.78	0.73	0.78	0.76
mean	0.7	0.72	0.76	0.76	

For this case, the average classification accuracy for all images was 0.74. For the considered combination of algorithms (SVM–MLP classifier, five best features, SFS selection method) we received the classification accuracy of 0.84 for the image P1, 0.69 for P2, 0.70 for P3, 0.71 for P4 and 0.77 for P5. The classification accuracies averaged for all tested methods were: 0.74 for the image P1, 0.64 for P2, 0.64 for P3, 0.66 for P4 and 0.72 for P5.

## Discussion

**Figure 2:**
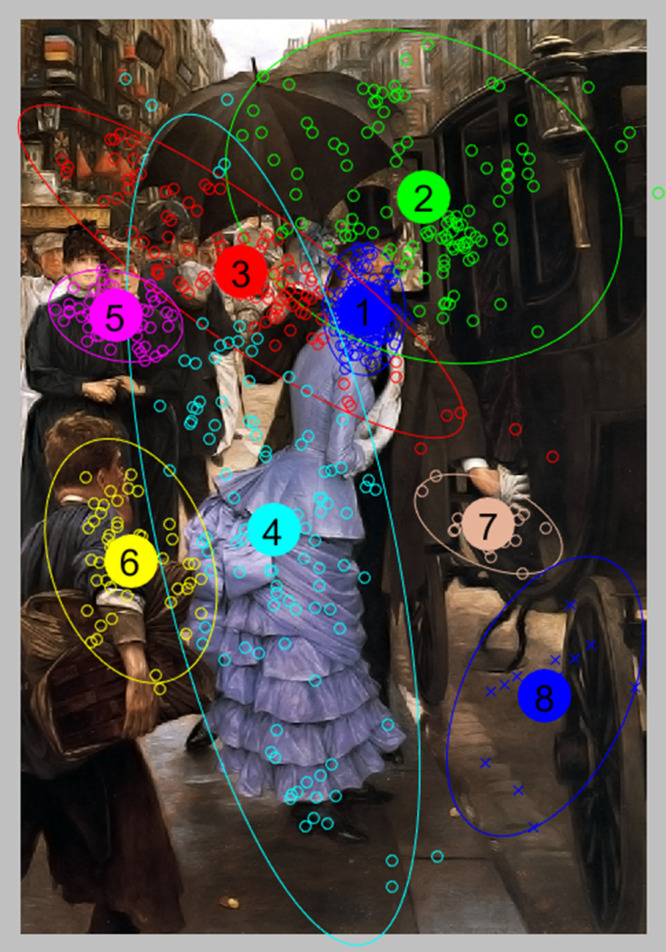
Result of clustering for EM method.

In Fig.2 the result of clustering with EM method is presented. The chosen number of clusters is eight. Each fixation belonging to the cluster is located near the center of gravity. EM algorithm allows you to create clusters using their statistical distributions. The omission of several fixation does not affect the determination of clusters as lack of some fixations does not disrupt calculation of statistical parameters [ 11 ]. The method of specifying the clusters has significant influence on further steps in quantitative description of a cluster. For example cluster #1 can be easily interpreted as being associated with a natural concentration of attention on woman’s face. Similarly, cluster #7 (brown) can be interpreted as associated with a concentration of attention on man’s hand. EM method takes into account statistical dependencies in the distribution of fixations and, to a large extent, allows the specification of clusters, which can be interpreted semantically. Very good results of implementation of mixtures of normal distributions can be obtained for clusters of elliptical shape. It was also found that the result of grouping using the EM algorithm is sensitive to the initial Θ_k_ parameter. For this purpose, the algorithm can be repeated many times for different initial parameters, and next, we can choose the best solution meeting the BIC.

We assumed that the method of data normalization could significantly affect the accuracy of classification. However, there was no such relationship. We did not find that normalization of data had a significant impact on classification accuracy. Average accuracies for the tested classifiers and different data normalized methods are presented in Table 8.

**Table 8.** Average classification accuracies for different data normalization methods.

Method	Z⁰	Z¹	Z²	Z³
Average accuracy	0.66	0.67	0.67	0.68

At classification stage, we used two kinds of features: number of fixations in a cluster and average fixation duration for a cluster. It was worth to find the feature which is a better to distinguish an expert from a nonexpert. For this purpose, we calculated the sum of t-values for all clusters of individual pictures (Table 9). It appeared that the better feature for distinguishing experts from non-experts was average fixation duration.

The average of the sum of t-values for individual images was 7.72 for the number of fixations and 13.12 for the average fixation duration (as features). The calculation of p-values and t-values was performed for data divided into two sets: experts versus non-experts. The p-values showed that calculated features for certain clusters enable to distinguish experts from the non-experts. For average fixation duration for the best cluster, there was no significant difference only for image P5 (p>0.05). The average p-value calculated for the best clusters of all images for average fixation duration was 0.03, whereas it was 0.26 for number of fixations. This confirms that better feature for distinguishing experts from non-experts is the average fixation duration than number of fixations.

**Figure 3:**
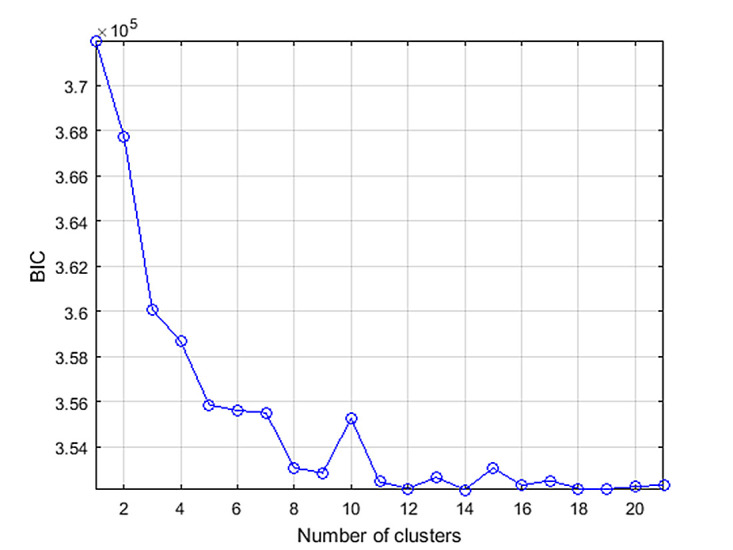
The dependence of the BIC value on number of clusters for the P2 image.

**Figure 4:**
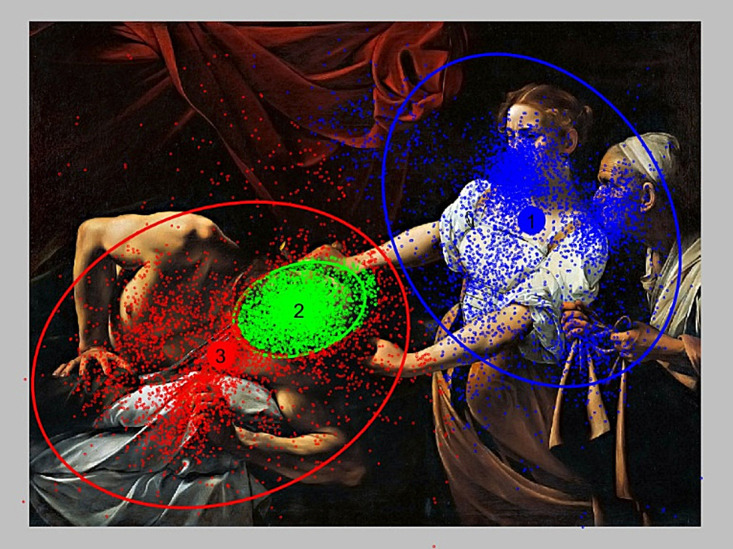
Fixation assignment to 3 clusters for the P2 image.

**Figure 5:**
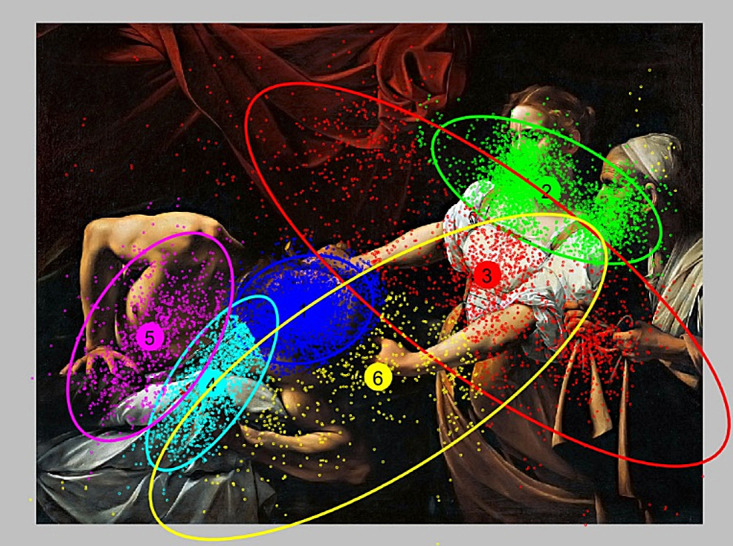
Fixation assignment to 6 clusters for the P2 image.

**Figure 6:**
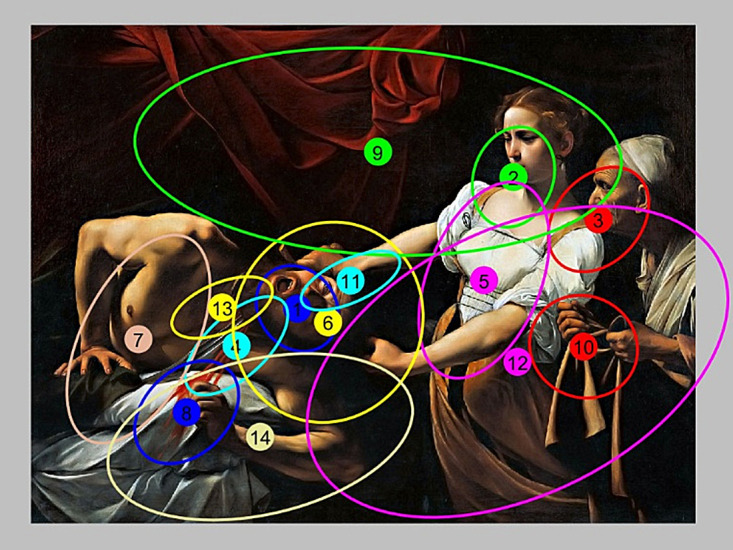
Ellipses for 14 clusters in P2 image.

An important element of the developed EM algorithm was assigning fixations to specific clusters and determination of the appropriate number of clusters. The list of optimal number of clusters for each image, calculated using BIC, is presented in Table 9. Proper cluster determination was significantly affected by the number of registered fixations. Too small number could be insufficient to calculate the representative clusters, which cover all ROIs. The dependence of BIC value on the number of clusters for P2 picture is illustrated in Fig. 3. In this case, the smallest (3.59×10^-5^) BIC value was for 14 clusters. The method of division fixations on clusters for different assumed number of them are presented in Figs. 4–6. For the case presented in Fig. 4, three clusters of fixations were created. It can be easily observed that it is not an optimum division. Intuitively, it should select more clusters in that case. Although cluster #2 represented fixations on one face, but still, there were not enough clusters representing the other faces.

**Table 9:** T-values, p-values and optimum number of clusters for the BIC criteria.

Parameters	P1	P2	P3	P4	P5	Mean
The sum of the coefficients t for the number of fixation	8.26	8.54	9.81	7.00	4.98	7.72
The sum of the coefficients t for the average fixation duration	16.35	13.65	13.08	10.96	11.54	13.12
The optimal number of clusters for the BIC criterion	11	14	12	12	12	12.2
P value for the best cluster for a number of fixation	0.114	0.071	0.853	0.229	0.047	0.26
P value for the best cluster for the average fixation duration	0.001	0.038	0.041	0.022	0.055	0.03

**Figure 7:**
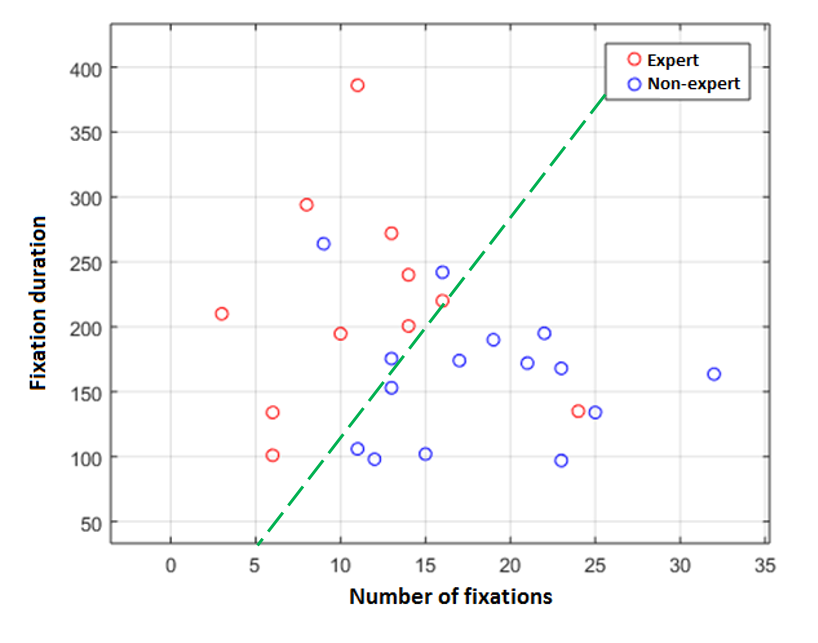
Distribution of the two features (number of clusters and average fixation duration for a cluster) for a group of experts and non-experts.

For the case of K=6 (Fig. 5), the situation improves, but still, the number of cluster is too small. Only for K=14 (Fig. 6), clusters could be interpreted as representative ROIs. Thus, the clusters #1, #2, and #3 can be interpreted as the ROI associated with the faces of individual characters. Cluster #4 is associated with a sword and so on. For greater clarity, Fig. 7 contains only ellipses of 14 clusters for P2 image.

Table 10 presents the average feature values (number of fixations) for experts and non-experts, and t-values calculated for each cluster of P2 image. It can be seen that there are two clusters for which the distribution of features suggests significant differences (p<0.1) in the group of experts and non-experts (cluster #1 and cluster #10). For cluster #1, the average number of fixations for experts was 13.6 and for non-experts 18.3 (p=0.07). The biggest statistical significance (p=0.04) was for cluster #10, for an average fixation duration as feature. For experts, the average fixation duration was 119.3 ms and for non-experts 128.1 ms. This is consistent with results obtained by other research groups.

**Table 10:** Average feature values (number of fixations) for experts and not of experts and t-value calculated for individual clusters designated for image P2.

	Cluster number													
	1	2	3	4	5	6	7	8	9	10	11	12	13	14
The average number of fixations: experts	13.6	10.9	4.9	4,6	3.9	3.5	3.3	3.15	2.55	1.45	1.35	0.85	0.7	0.65
The average number of fixation: non-experts	18.3	9.4	6.58	5.26	4.47	3.89	3.58	2.47	2.32	1.95	1.79	0.74	0.68	0.37
p for the number of fixations	0.07	0.37	0.17	0.47	0.72	0.76	0.76	0.61	0.75	0.47	0.45	0.80	0.96	0.45
Mean fixation time: experts	214.3	192.1	178.8	218.8	117	164.4	166.8	126.4	143.8	119.3	128.0	44.9	60.7	44
Mean fixation time: non-experts	191.7	167.9	185.1	171.2	158.7	184.2	117.1	101.1	108.3	128.1	128.1	50.6	54.5	18.7
p for fixation time	0.33	0.11	0.56	0.14	0.77	0.75	0.18	0.21	0.10	0.04	0.30	0.52	0.98	0.42

Krupiński [ 26 ] and Manning [ 27 ] showed that in comparison to non-experts, experts typically perform tasks with fewer fixations. At works [ 28,29 ] it was shown that experts had longer fixation durations than novices when driving a car.

The distribution of the number of fixations (cluster #1) and the average fixation duration for cluster (cluster #1) for group of experts and non-experts is shown in Fig. 7.

Clusters calculated for all P1–P5 images using EM methods and BIC are given in Supplementary Materials (available online).

## Conclusions

The proposed algorithm allows us to automatically classify a person watching a painting to a group of experts or not-experts in the field of art. A key role in the proposed algorithm is EM clustering method. With this method it is possible to determine ROIs on the image. With features selected for the ROIs, such as: number of fixations and average fixation duration, and automatic classification of an image viewer is possible. The algorithm was tested in such a way as to get close to the actual conditions of operation of the expert system.

### Ethics and Conflict of Interest

The authors declare that the contents of the article are in agreement with the ethics described in http://biblio.unibe.ch/portale/elibrary/BOP/jemr/ethics.html and that there is no conflict of interest regarding the publication of this paper.

### Acknowledgements

This work was supported in part by the grant of National Science Centre (Poland) No. DEC2013/11/B/HS6/01816.
